# Nanoscale Observation of Dehydration Process in PHEMA Hydrogel Structure

**DOI:** 10.1186/s11671-017-2055-3

**Published:** 2017-04-26

**Authors:** Kordian Chamerski, Witold Korzekwa, Jacek Filipecki, Olha Shpotyuk, Marcin Stopa, Piotr Jeleń, Maciej Sitarz

**Affiliations:** 1 Institute of Physics, Faculty of Mathematics and Natural Science, Jan Dlugosz University in Czestochowa, Al. Armii Krajowej 13/15, 42-200 Czestochowa, Poland; 2 Komed Clinic, Sobieskiego 54, 42-200 Czestochowa, Poland; 3 Danylo Halytsky Lviv National Medical University, 69, Pekarska str., Lviv, 79010 Ukraine; 4 Department of Optometry and Biology of Visual System, Poznan University of Medical Sciences, ul. Rokietnicka 5D, 60-806 Poznan, Poland; 5 Clinical Eye Unit and Pediatric Ophthalmology Service, Heliodor Swiecicki University Hospital, Poznan University of Medical Sciences, Poznan, Poland; 6 AGH University of Science and Technology, Faculty of Materials Science and Ceramics, Al. Mickiewicza 30, 30-059 Krakow, Poland

**Keywords:** Dehydration, FTIR, Hydrogen bonding, PALS, Free volume, PHEMA

## Abstract

One of the most important field of interest in respect to hydrogel materials is their capability to water storage. The problem mentioned above plays an important role regarding to diffusion of fluid media containing nanoparticles, what is very useful in biomedical applications, such as artificial polymeric implants, drug delivery systems or tissue engineering.

In presented work, dehydration process in hydrogels used in ophthalmology as intraocular lenses was observed. Before measurements studied materials were immersed in deionized water and saline solution to obtain equilibrium swelling state. Studies of the dehydration process were carried out by use of gravimetric analysis, Fourier-Transform Infrared and Positron Annihilation Lifetime Spectroscopy. Obtained results revealed changes in hydrogen bonding structure and free volume holes induced by saline solution ingredients.

## Background

The state of biomaterials science has developed greatly over the last few decades. Development of technology as well as greater care for human life and health have contributed to this state of knowledge. Current research on biomaterials is mostly focused on the development and improvement of materials used as drug delivery systems, scaffolds or artificial implants. Biocompatible materials, such as metals, glass-ceramics, polymers, and composites, are some of the materials commonly used for biomaterials production [1].

All kinds of biomaterials used as implants are produced with the aim of replacing and taking over the function of natural biological tissues which have been damaged or removed. In such a context, biomaterials are often called functional materials and their function is to improve the comfort or even sustain human life [2]. Undeniably one of the fields of medicine in which these types of functional materials are commonly used is ophthalmology. One of significant applications is using biomaterials for correction and restoration of sight with the use of contact and intraocular lenses. Nowadays, soft acrylic polymers, hydrogels, and silica hydrogels are mostly used in production of such lenses [3]. These materials usually show good biocompatibility and functionality; however, due to their constant contact with aqueous humor and any other ingredients artificially introduced to the eyeball, it may happen that properties of the materials will change, what will prevent proper functioning of the lens [4–7]. The abovementioned reasons suggest the necessity of conducting the studies in the field of environmental influence on the biomaterials properties. The transport of fluids containing different kinds of chemical compounds through the hydrogel structure plays an important role in the case of hydrogels application as scaffolds or drug delivery systems [8], thus the mass transport through the lens material must be also taken into account.

Poly(2-hydroxyethyl methacrylate) (PHEMA) is the generally used hydrogel material in intraocular lenses (IOLs) production. Since the PHEMA material possesses hydrophilic OH and C = O groups capable of formation of hydrogen bonds with water molecules and themselves, the studies on hydrogen bonding structure both in dry [9] and wet [10] materials were conducted. The mentioned studies revealed the differences in hydrogen bonding structure in both cases in the basis of vibrational spectroscopy. Studies performed on many other hydrophilic materials conducted by means of Fourier-Transform Infrared (FTIR) technique have revealed the existence of two kinds of OH groups in the structure, namely hydrogen and non-hydrogen bonded [11–13]. Furthermore, the case of hydrogen bonds formed in hydrophilic structures is more complex due to inter- and intramolecular bonds existence [14, 15]. As it can be seen, studies performed by use of FTIR method give the detailed information about molecular bonding at the molecular level. Thus, studies focused on the impact of liquid media containing chemicals on the molecular structure can be observed in the basis of hydrogen binding structure changes, especially since the influence of both some salts solution on molecular structure in liquid water [16] and glucose on enhancing stability of hydrogels [17] were observed. Moreover, the support of above approach by use of Positron Annihilation Lifetime Spectroscopy (PALS) could provide the information related to the free volume holes changes associated with structural bonding transformation.

The objective of this work was an observation of the dehydration process in hydrogel polymeric material used as IOL device. The transport process was discussed and analyzed on the ground of structural changes related to hydrogen bonding and free volume holes. Results obtained in this study provide the knowledge in the field of inorganic ingredients impact on hydrogel structure and mass transport by such a material.

## Methods

### Materials

One model of commonly used polymeric intraocular lens (IOL), investigated in previous study [18], was investigated in this study. Flexible IOL Quatrix (CROMA –PHARMA GmbH, Leobendorf, Austria) with water content equal to 26%, based on poly(2-hydroxyethyl methacrylate) (PHEMA) hydrogel and characterized by 0.5 mm of thickness was applied as research material. Each sample material was provided in its original packaging in a 0.9% saline solution bath. Before measurements all samples were immersed in deionized water to rinse the saline ingredients from the internal structure of materials. During the immersion the deionized water was changed every 2 h for the first 10 h. Then, samples were put in deionized water for the next 14 h to achieve fully swollen state before experimental analysis. After first set of dehydration measurements, the same dried samples were put in saline solution for the 24 h to achieve fully swollen state by saline solution. In the next step, the dehydration process was observed for so prepared samples. Swelling process for both deionized water and saline solution was performed in temperature in the range of 22–24 °C.

### Gravimetric Analysis

For gravimetric observation of dehydration process in prepared samples, the analytical balance with the accuracy equal to 0.1 mg was applied. The time of measurement stabilization was estimated to be up to 5 s.

Each sample was pulled out from the fluid before measurements and dried by use of tissue paper to drain excess of moisture from the material surface. Then, samples were weighted at the analytical balance every 30 min, except first 14 min of dehydration process when materials were weighted every 2 min to obtain more precise data of mass loss in the beginning of the process. The whole measurement procedure was performed within 24 h in ambient temperature 22–24 °C.

As a result of gravimetric measurements the dehydration curves were obtained. Each dehydration curve was recorded as the changes in the mass ratio *M*_*t*_ */M*_*∞*_ of investigated sample in the function of square root of dehydration time, where *M*_*t*_ is the mass of the water amount in the time *t* and *M*_*∞*_ is the mass of the water content in fully swollen sample. Mass ratios were acquired by the following equation:

1 $$ \frac{M_t}{M_{\infty }}=\frac{m_t-{m}_d}{m_{\infty }-{m}_d}, $$

where *m*_*t*_ means the mass of the sample in the time *t* of dehydration process, *m*_*∞*_ is the mass of the sample in the equilibrium swelling state and *m*_*d*_ is the mass of the dried material [19]. If the (1) is known for values near the 0.5, the calculation of diffusion coefficient is possible as follows [20]:

2 $$ D=\frac{\pi}{16}{\left(\frac{M_t/{M}_{\infty }}{\sqrt{t}/ L}\right)}^2 $$

where *D* is the diffusion coefficient measured in [cm^2^ · min^−1^], *t* is the time of measured mass loss, and *L* is the sample thickness. Coefficient acquired by means of (2) equation is a measure of diffusion rate of solution filling the pores of the material.

### Fourier-Transform Infrared (FTIR) Measurements

FTIR experiment was conducted by means of Attenuated Total Reflectance (ATR) method. The Bruker Tensor 27 spectrometer was used as analytical device. Measurement procedure performed to observe of dehydration process in investigated samples lasted within 1440 min. At this time the set of infrared spectra in the range of 500–4000 cm^−1^ was obtained. Each spectrum was acquired in air condition with the 4 cm^−1^ resolution and consisted of 32 scans to shorten the acquisition time.

Dehydration analysis was performed by observation of OH stretching band changes. The continuous band occurring in the range of frequencies between 3000–3700 cm^−1^ for each sample was fitted to theoretical one. The fitting procedure assumed application of Lorentzian and Gaussian functions with 50:50 proportion and four components fitting [21]. Component with the frequency over 3600 cm^−1^ was assumed to be connected with non-hydrogen bonding OH stretching vibrations whereas three components in the range of 3200–3600 cm^−1^ was interpreted as hydrogen bonded OH stretching vibrations.

### Positron Annihilation Lifetime Measurements

Free volume evolution during dehydration process was observed by means of Positron Lifetime Spectroscopy (PALS). The Ortec positron lifetime spectrometer with the 300 ps resolution was used to obtain lifetime spectra. Resolution of apparatus was acquired as the FWHM in ^60^Co gamma isotope measurements. The ^22^Na isotope with the starting activity equal to 4 · 10^5^ Bq closed between two pieces of kapton foil was applied as the positron source. More detailed description of apparatus was published elsewhere [22].

Observation of dehydration process by PALS method was performed at the 22 °C of temperature and 30–40% of humidity. In the first step, two identical samples are pulled out from the fluid and dried at the surface. Subsequently, samples are closed between two pieces of kapton foil with the thickness equal to 25 · 10^−6^ m and subjected to positron lifetime analysis. Kapton foil was applied to slow down of dehydration process and observe the internal structure in the state close to fully swollen. After 24 h, both samples were pulled out from kapton foil and immersed in fluid to get equilibrium swelling state. So, prepared samples were then be subjected to positron lifetime measurements without kapton foil until be dried.

As a result of PALS measurements, the positron lifetime spectra were obtained. Each spectrum was subjected to analysis by use of LT 9 computer program [23] with the three component fitting procedure. First component τ_1_, was assumed to be parapositronium (p-Ps) lifetime with constant value 125 ps, second component τ_2_, was interpreted as free annihilation of positrons from localized and delocalized states in structure and third component τ_3_,was taken into account as orthopositronium (o-Ps) lifetime in free volume holes. Analysis of free volume holes was performed by application of Tao-Eldrup model describing the relationship between o-Ps lifetime and spherical free volume hole radius as follows:

3 $$ {\tau}_3=0.5{\left[1-\frac{R}{R+\varDelta R}+\frac{1}{2\pi} \sin \left(\frac{2\pi R}{R+\varDelta R}\right)\right]}^{-1} $$

where *ΔR = 0.166 nm* is the thickness of electron layer at the wall of free volume hole [24–26]. The spherical free volume hole size was calculated by means of simple relation:

4 $$ V=\frac{4}{3}\pi {R}^3 $$

## Results

**Fig. 1:**
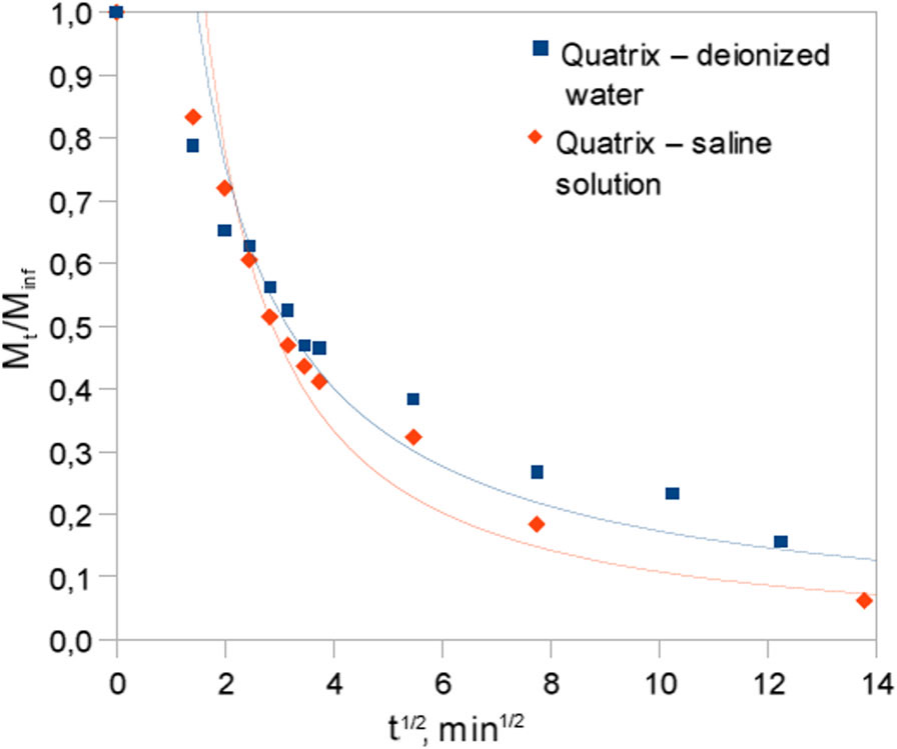
Dehydration curves obtained as changes in mass ratios

In the first step, gravimetric analysis of immersed samples was performed. As a result of measurements, two dehydration curves were obtained as a changes of mass ratios in the function of square root of time. Figure 1 shows dehydration curves both for sample subjected to pure water bath and for sample immersed in saline solution. Acquired curves indicate the same progress of dehydration process in both samples. However, the rate of dehydration is slightly higher for sample containing saline solution and the water content for such sample is lower at the end of the process. Chart was limited to first 200 min (~14 min^1/2^) of the process due to demonstrate the initial stage of desorption in a clear manner. On the basis of results obtained by gravimetric analysis the diffusion coefficients for both deionized water and saline solution were calculated. Results of calculations performed for M_t_/M_∞_ = 0.47 are presented in Table 1. Gravimetric measurements of diffusion properties indicate the increasing of diffusion coefficient *D* in sample containing saline solution.

**Table 1:** Results of diffusion coefficient calculations

Solution	M_t_/M_∞_	t^1/2^ [min^1/2^]	L [cm]	D [cm^2^ · s^−1^]
Deionizedwater	0.47	3.46	~0.5	(1.498 ± 0.005) · 10^−7^
Saline		3.16		(1.798 ± 0.005) · 10^−7^

**Fig. 2:**
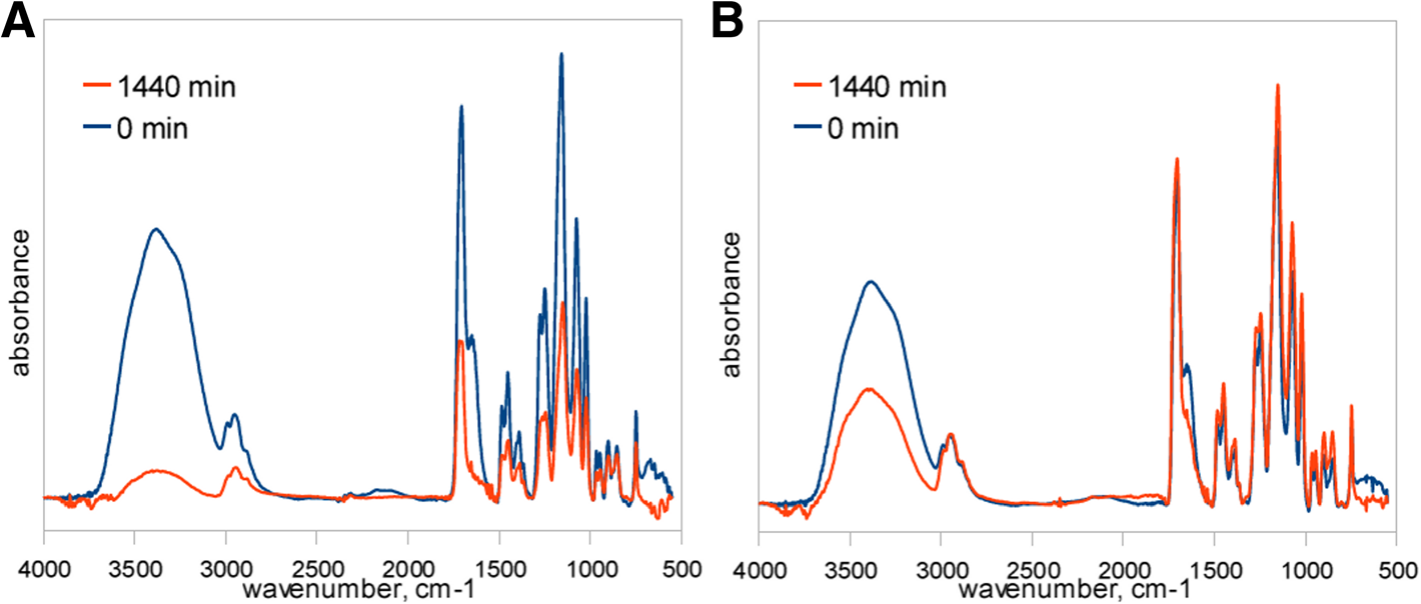
FTIR spectra of material subjected to (**a**) deionized water and (**b**) saline solution

**Fig. 3:**
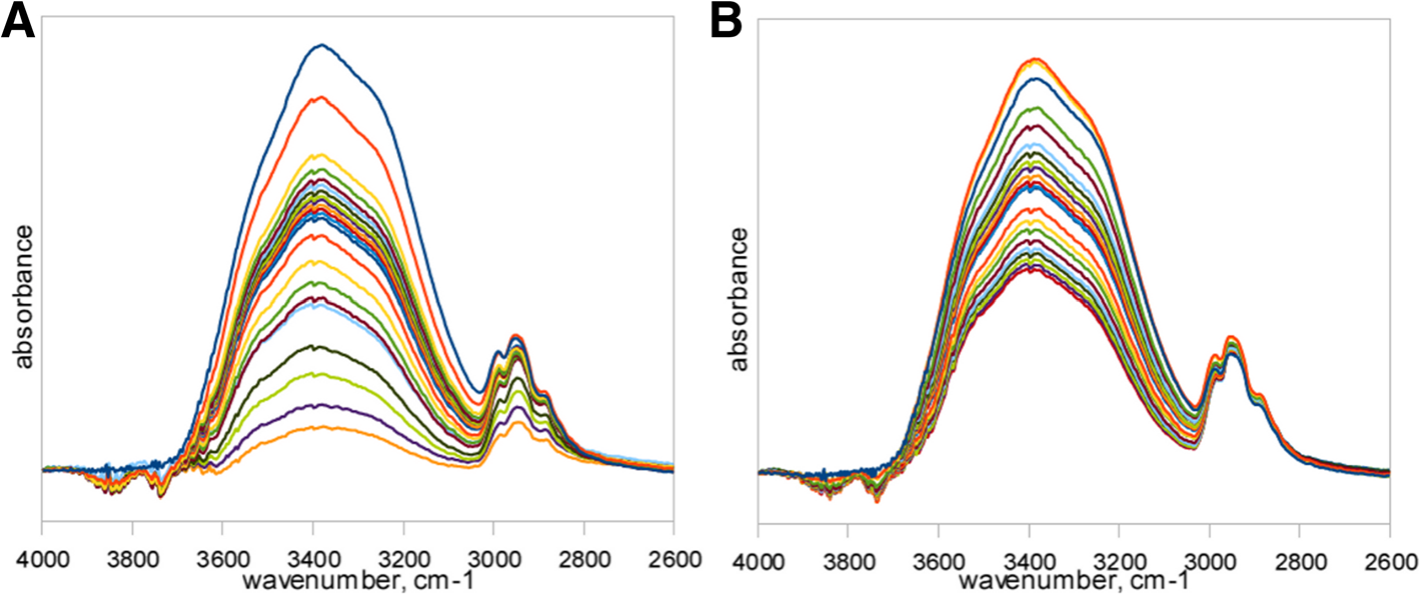
OH stretching band changes in (**a**) deionized water and (**b**) saline solution subjected samples

**Fig. 4:**
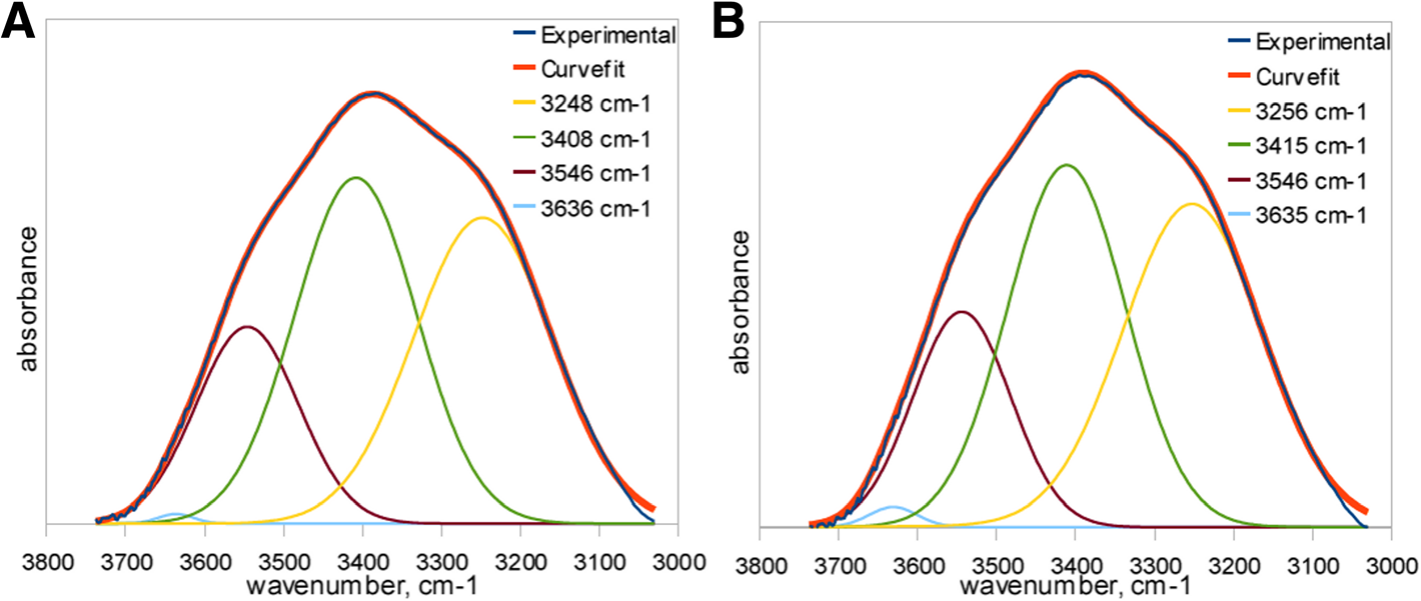
Demonstration of OH sub-bands fitting procedure for (**a**) deionized water and (**b**) saline solution

Much more complex was the analysis by use of FTIR ATR method. Figure 2 presents spectra of sample subjected to deionized water bath (Fig. 2a) and sample held in the saline solution (Fig. 2b). Both diagrams show spectra at the start and the end of dehydration process in the full range of frequencies. The continuous OH stretching band at the range of 3000–3700 cm^−1^ was used for hydrogen bonding structure analysis. In the Fig. 3, the dehydration process on the basis of OH stretching band changes was presented. Above spectra show the changes in the integral intensity of OH band and accompanying slight shift of band towards the higher frequencies in the range of 0–1440 min of time with spectra acquisition every 30 min. Further band analysis was performed by the fitting procedure described in previous section. Figure 4 presents results of fitting for samples immersed in both pure water and saline solution at the start of dehydration process. The best fitting was obtained with four sub-bands. The band in the range of 3630–3670 cm^−1^ with the lowest intensity indicates non-hydrogen bonded OH groups existed in the samples, whereas three other bands at frequencies about 3240–3280, 3400–3450, and 3540–3550 cm^−1^ are the evidences of hydrogen bonds with different degree of bonding. Table 2 shows frequencies of the considered OH bands and kinds of hydrogen bonds assigned for them [9].

**Table 2:** Assignments of considered IR bands in PHEMA

Assignments	Wave number, cm^−1^
Free OH	3630–3670
Dimer OH (OH · · · OH), intramolecular OH (OH · · · O = C)	3540–3550
Intermolecular OH, Aggregates OH (· · ·OH · · · OH · · · OH · ··)	3400–3450
	3240–3280

**Fig. 5:**
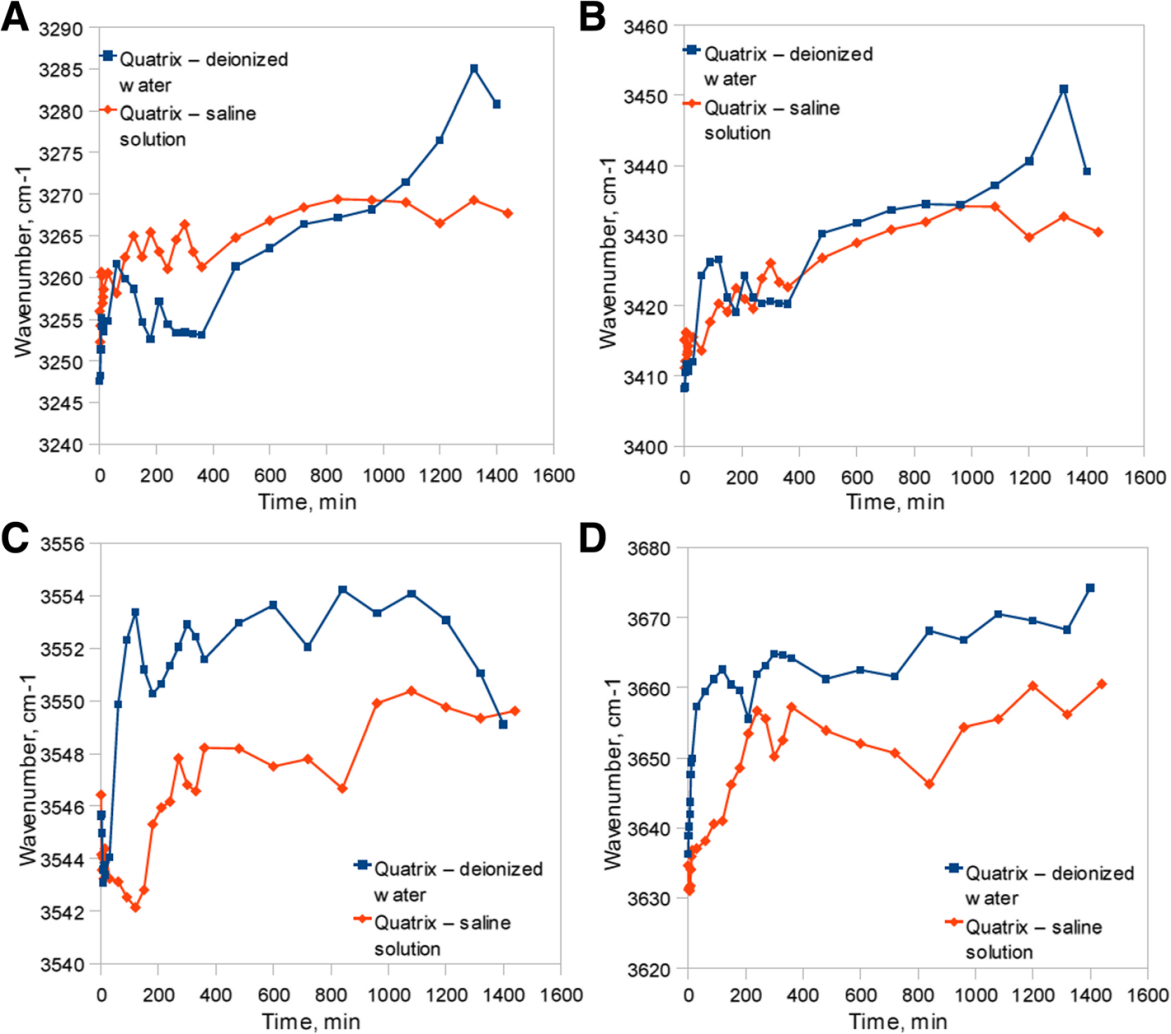
Shifting of (**a**) 3250 cm^−1^, (**b**) 3400 cm^−1^, (**c**) 3545 cm^−1^, and (**d**) 3635 cm^−1^ OH stretching sub-bands in time

**Fig. 6:**
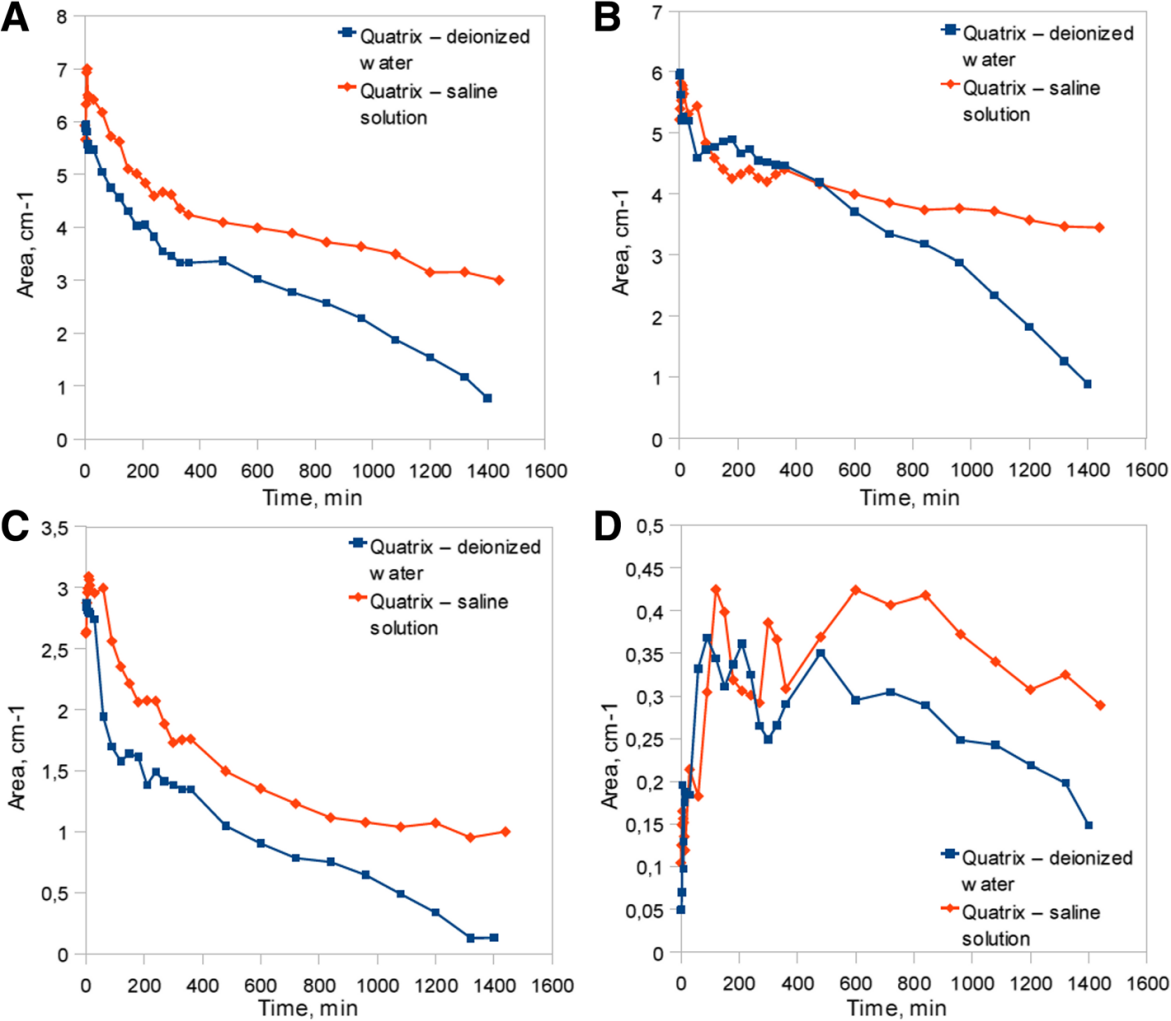
Integral intensity changes in time for OH (**a**) 3250 cm^-1^, (**b**) 3400 cm^-1^, (**c**) 3545 cm^-1^ and (d) 3635 cm^-1^ sub-bands

The band shifting gives the information about bonds strength and indicates on bond strength increasing with the increasing of bands frequency. Figure 5 shows the bands shifting during the dehydration process for samples. Both for the pure water and saline solution sample the shifting towards higher frequencies can be noticed. However, band shifting is more slight for sample containing the saline solution. The integral intensity is the other interesting parameter to analyze the H-bonding changes in the internal structure during dehydration process. Figure 6 presents the integral intensity changes described as band area in the function of dehydration time. Despite that the decreasing of band area for each H-bonded sub-band is observed, the integral intensity for saline solution subjected samples is diminished with lower rate.

**Fig. 7:**
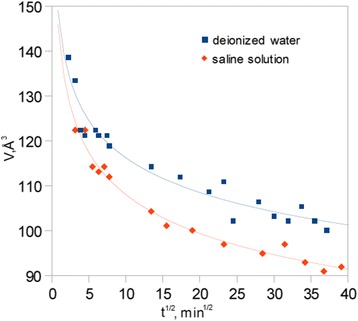
Free volume holes evolution during dehydration process

**Fig. 8:**
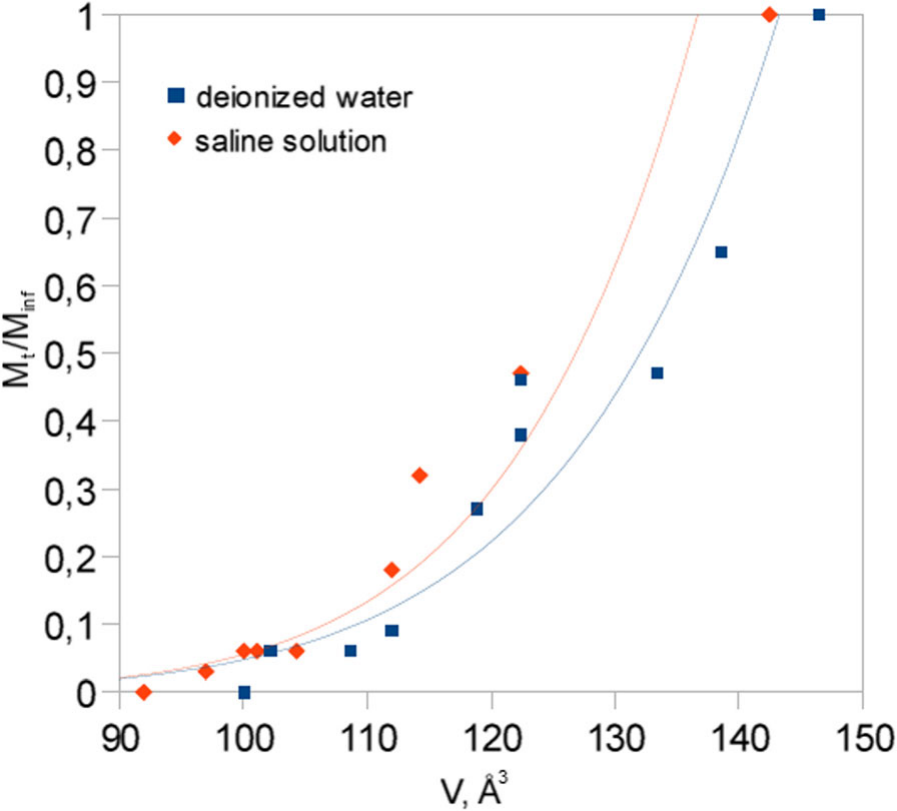
Relationship between relative water content and free volume holes sizes

Positron Annihilation Lifetime Spectroscopy was used as last method for dehydration process observation. As a result of measurements, the dehydration curves were acquired as the changes of free volume holes dimensions in the function of dehydration time. Figure 7 presents the diagram with dehydration curves for both pure water and saline solution subjected samples. The lifetime of o-Ps is decreased with time, what indicates on free volume holes evolution towards their reduction in size. Moreover, it has to be noticed that free volume sizes are smaller during the dehydration process for samples subjected to saline solution. Dependence between free volume evolution and water content in studied materials is presented in Fig. 8. Such an analysis indicates the impact of saline ingredients on water storage in hydrogel materials. More results analysis is presented in the next section.

## Discussion

Observation of dehydration process on both the macroscopic and molecular level in polymeric hydrogel structures subjected to bath in different fluids was the aim of presented study. Achievement of the aim pursued was performed by application of gravimetric measurements rely on track changes in hydration of samples and support of obtained results in such a way by observation of free volume and hydrogen bonding changes in the internal structure during the dehydration process.

Since the hydrogel structure exists in equilibrium swelling state only with bath solution, pulling of the material from the fluid causes diffusion and evaporation of liquid media filling its internal structure to compensate the concentration of fluid molecules inside and outside of the material. During such a process, the investigated sample loses weight in time. Well known is the fact that water effects on internal structure of PHEMA hydrogel as a plasticizer, thus decrease of its amount inside the sample makes the structural changes of the material in phase transition occur. Changes described above can appear in molecular bonding properties and distances between molecules, what can be observed in vibrational spectra of molecules and free volume evolution measurements, respectively.

Gravimetric analysis carried out at first step of investigation has given the information about the transport rate due to desorption process of fluid filling the material structure. Two parts on curves describing progress of dehydration process (Fig. 1.) can be distinguished, first, 20 min lasting and characterized by higher slope and second, much longer lasting with smaller slope. The first one, representing transport of free water in hydrogel structure, is useful for diffusion coefficient determination. Fick’s diffusion model can be used in this case. The sample subjected to saline solution exhibits slightly higher rate of free water diffusion incipient of the process than pure water immersed sample, what is mirrored in higher value of diffusion coefficient. It is in opposite to the conclusion that salts retain water at internal biological structures. Observed result is due to either influence of saline ingredients on water molecules to make less complex clusters leave the structure or effect of dehydration history caused by subject of sample to several processes of structure dehydration. Former effect can be explained with respect to the physical meaning of diffusion coefficient. Well known is the fact that diffusion coefficient is inversely proportional to the cross section of diffusing molecules. Thus, increasing of *D* can be the indication of decreasing of water clusters size influenced by saline ingredients. A similar problem was observed for water liquid solutions containing salts. In the field of flotation chemistry, the influence of ions of salts on water structure is commonly investigated [16]. According to this field of science, the structure of water in such solutions can be well organized or disturbed depending on kind of dissolved salt, what results in existence of ice-like water and liquid-like water structures, respectively. In the case of impact of deionized water on the studied samples, the water structure can be considered as ice-like due to lower diffusion of solute through internal structure of material. The above conclusion can be explained by the existence of stronger hydrogen interactions between water molecules in such a system or influence of surrounding polymer network on water filling the pores. In turn, the water structure in the sample influenced by saline can be assumed to be liquid-like due to higher mobility of water molecules what results in higher diffusion rate of such solution. The second problem mentioned above, which may have the impact on diffusion process, dealing with material dehydration history, is well known in biomaterials science and leads to decrease of water content in the internal structure at equilibrium swelling state. Therefore, the polymer network could be changed and resulted in the differences of calculated diffusion coefficients rather than impact of solutes on water structure. Since the investigated sample was immersed in pure water and dehydrated before the saline solution immersion, such a problem has to be taken into account.

Analysis of the water structure in the studied material has given the information about strength of hydrogen bonds between water molecules in hydrogel structure. Results presented in Fig. 5 indicate that up to 100 min of dehydration process large shifts in each OH band for deionized water occur. Above observation is connected with the fact of water desorption what leads to less interactions between water molecules and thus stronger oscillation frequency of remaining OH bonds. After 100 min the shift of OH bands for pure water is lower, what could be assigned for the transition of water structure existing in material. Such an effect may be interpreted in the basis of three state model of water structure. According to the three state model, water molecules can exist in three kinds of states in the hydrogel structure, namely bounded (hydration) state characterized by presence of hydrogen bonds between water and hydrophilic as well as hydrophobic groups in polymer network, interstitial state, where water molecules are surrounded by polymer network and free state also called as bulk water [27–29]. Band shift existing up to 100 min probably reflects in desorption of bulk water and is connected with appearance of high amount of non-bonded water molecules what can be presented by increase of integral intensity of 3600 cm^−1^ band in Fig. 6d. Between 100–1000 min process of interstitial water desorption exists what can be observed in shift of bands assigned to both intermolecular (Fig. 5a, b) and dimer (Fig 5c) OH bonds. Subsequently, above 1000 min, last transition of water structure takes place, what can be an indication of bound water desorption. Analysis of integral intensities (Fig. 6) shows decrease in intensity of each band connected with OH bonds. In the case of sample containing saline solution, the lower shifts towards higher frequencies for hydrogen bonds are observed. Presence of the only first transition in water structure can be noticeable what leads to conclusion of water retention in the hydrogel structure by saline ingredients. Above observation can be in conformity with changes in bands intensity, which decrease up to 800 min and then is approximately constant, what indicates the lack of bounded water desorption. Therefore, it can be concluded that the saline ingredients have an impact on creation of hydrogen bonds inside the material leading to the formation of more stable water structure connected by more durable bindings than it is in pure water.

The above results are consistent with PALS measurements of the free volume holes evolution during dehydration process. Since the PALS method gives the information concerning of o-Ps lifetime in empty intermolecular spaces, thus curves presented in Fig. 7 show the changes in size of empty area inside the pores filled by water or saline solution. In the beginning of the process, the material pores are fully filled by solution what indicates the structure near the equilibrium swollen state. Such a state near the equilibrium state is represented by the first measure point at both curves. Smaller free volume holes, connected with empty spaces between molecules filling the pores, are noticed in the case of saline solution. However, such an effect is particularly seen in the end of the dehydration process where free volume sizes should be the same for both saline and deionized water impacted samples in dried state. More hydrogen bonded structure observed in IR experiment results less free space for localization of o-Ps, what translates to smaller sizes of free volume holes in the case of saline solution influence. In turn, the beginning of the free volume evolution is in agree with gravimetric analysis, namely higher rate of free volume evolution on approximated curves is observed in the case of samples containing saline solution due to higher rate of diffusion of water clusters impacted by saline ingredients. Therefore, dual effect of saline solution can be distinguished. Firstly, the ingredients of salts act as water structure breakers, on the other hand their impact is noticeable as water structure makers which can retain water in the hydrogel structure. Since saline solution consists of multiple salts and ingredients, the above problem is difficult to explain. Thus, more extended studies on the impact of individual salts on hydrogel structure should be conducted. According to the three state model of water structure, gravimetric analysis and PALS experiment do not give the information on water molecules tightly bounded with polymer network. In contrast to OH bands analysis performed by FTIR method, in other methods the transition of bound water cannot be observed. It is just because the bounded water is the small amount of whole water content in the sample and it is difficult to observe its desorption during gravimetric experiment. The transition of bounded water desorption could be observed by increase the frequency of measurements both in gravimetric analysis as well as PALS experiment and should be the aim of further studies.

Extremely interesting become the relation between water amount and free volumes observed during dehydration process. Curves presented in Fig. 8 can give the evidence of water retention in the sample impacted by saline solution. The water amount in the sample treated by saline is always higher for a given free volume hole size than in the case of deionized water. Since obtained curves are only approximated, consequently greater quantity of measure points should be obtained in order to get better curves fitting to the experimental data. Such approach should give the clearer results and will be the subject of further studies.

Analysis of dehydration process in hydrogel materials is an extremely difficult task. Observation of the process at the molecular level has given the information about water transport in the free volume holes on the basis of changes in hydrogen bonds and demonstrated more filled and hydrogen-bonded structure in the case of fluid containing inorganic compounds. More stable network formation can be explained by influence of such compounds on changes in water binding, and thus in internal structure transformation towards its improvement.

## Conclusions

Dehydration process in polymeric hydrogel IOLs subjected to the deionized water and saline solution influence was conducted in this study. Problem of transport in hydrogel structures is very important in the biomaterials science point of view. Disturbances in proper mass transport in IOL materials can lead to deterioration of functional properties for such medical devices, thus studies on the field of mutual interactions between the implant materials and environment components have to be performed. In the presented study, the influence of saline solution on the internal structure of IOL was demonstrated on the basis of the free volume holes evolution and hydrogen bonding structure changes during the transport process. Studies revealed the existence of more complex hydrogen bonding structure in the case of saline solution immersion, what in turn has the impact on the free volume holes structure. More hydrogen-bonded structures exhibit less of empty area inside the material, what affects on mass transport. In the case of saline solution, it could be advantageous in view of probably diffusion attenuation of aqueous humor ingredients which impact could be undesirable. However, the influence of other, potentially bothersome inorganic compounds, such as calcium phosphates, has to be also studied.

## Abbreviations

ATR: Attenuated Total Reflectance
FTIR: Fourier-Transform Infrared
FWHM: Full Width at Half Maximum
IOL: Intraocular lens
LT: Lifetime programme
PALS: Positron Annihilation Lifetime Spectroscopy
PHEMA: Poly(2-hydroxyethyl methacrylate)
